# Crowdsourced tick observation data from across 60 years reveals major increases and northwards shifts in tick contact areas in Finland

**DOI:** 10.1038/s41598-023-48744-8

**Published:** 2023-12-02

**Authors:** Jani J. Sormunen, Ilari E. Sääksjärvi, Eero J. Vesterinen, Tero Klemola

**Affiliations:** 1 Biodiversity Unit, University of Turku, Turku, Finland; 2 Institute of Biology, University of Neuchâtel, Neuchâtel, Switzerland; 3 Deparment of Biology, University of Turku, Turku, Finland

**Keywords:** Ecology, Public health

## Abstract

There is mounting evidence of increases in tick (Acari: Ixodidae) contacts in Finland during the past few decades, highlighted by increases in the incidence of Lyme borreliosis and tick-borne encephalitis (TBE). While nationwide field studies to map distributions of ticks are not feasible, crowdsourcing provides a comprehensive method with which to assess large-scale changes in tick contact areas. Here, we assess changes in tick contact areas in Finland between 1958 and 2021 using three different nationwide crowdsourced data sets. The data revealed vast increases in tick contact areas, with ticks estimated to be contacted locally approximately 400 km further north in western and approximately 100 km further north in eastern Finland in 2021 than 1958. Tick contact rates appeared to be highest along the coastline and on the shores of large lakes, possibly indicating higher tick abundance therein. In general, tick observations per inhabitant increased from 2015 to 2021. Tick contact areas have expanded in Finland over the past 60 years. It appears that taiga ticks (*Ixodes persulcatus*) are behind most of the northwards shifts in tick contact areas, with *Ixodes ricinus* contributing mostly to new contact areas in the south. While ticks are now present in most of Finland, there are still areas where tick abundance is low and/or establishment not possible, mainly in northern Finland.

## Introduction

Ticks and tick-borne diseases (TBDs) form a significant and growing threat to human health and well-being in Europe, with hundreds of thousands of cases of TBDs reported annually^1^. The most commonly reported TBD in Europe is Lyme borreliosis, caused by *Borrelia burgdorferi* sensu lato spirochetes^1^. The occurrence and prevalence of *B. burgdorferi* s.l. is relatively uniform among populations of its main vectors, i.e., nymphal and adult ticks belonging to the *I. ricinus* species complex^2,3^, so increases in abundance or geographical distribution of *Ixodes* ticks are likely to be mirrored rapidly in disease cases. It has been estimated that the ongoing climate change will significantly influence the life cycles of both ticks and tick-borne pathogens, leading to rapid changes in tick distribution areas, tick abundance in established areas and prevalence of tick-borne pathogens^4,5^.

The effects of climate change on tick populations are dependent on the local climate, geography and tick species present^6^. For Finland, the closest environmental analogues are neighboring Sweden, Norway and European parts of Russia, where the same generalist tick species (*I. ricinus* Linnaeus, 1758 and *I. persulcatus*, Schulze, 1930) are present and the effects of climate change may be expected to be similar. Studies have revealed latitudinal (northwards) and altitudinal shifts in geographical distribution and increases in abundance for *I. ricinus* in Sweden and Norway during the past decades^7,8^, as well a similar changes for *I. persulcatus* in European parts of Russia^9,10^. There is also some indication of local increases in tick abundance in the southern parts of Finland^11–13^. Furthermore, cases of Lyme borreliosis and tick-borne encephalitis (TBE) have increased during the past few decades^14–16^, including more cases from the western and northern parts of Finland, suggesting increases in tick abundance and shifts in distribution ranges.

While historical field survey data is lacking in Finland, the nationwide geographical distributions of the local human-biting tick species have been mapped through citizen science. The first mapping was conducted in the late 1950’s^17^. During this time, all observed ticks were estimated to be *I. ricinus*. The author conducted field surveys in eastern Finland and the Åland Islands between Finland and Sweden, but identified only *I. ricinus* among collected ticks. However, as nationwide distribution was otherwise assessed based on answers to questionnaires sent by letter, the presence of other *Ixodes* species could not be ruled out. In any case, the incidence of dysentery in cattle was also observed to correspond to the reported areas of common tick presence, offering credibility for the assessments of tick distribution and abundance^17^.

The next nationwide study of tick distribution was conducted in two phases over 50 years later, in 2014–2015, when researchers from the University of Turku asked people to report their tick sightings in a web questionnaire (in 2014)^18^ and then (in 2015) to send ticks they found by letter for identification and pathogen analyses^19,20^. The latter study phase revealed that *I. persulcatus* was now present in vast areas of the country, even appearing to be the dominant species in some areas, particularly the western coast^20^.

The latest nationwide crowdsourcing campaign, Punkkilive (www.punkkilive.fi/en), was launched in 2021. Punkkilive (“*ticklive*”) is an interactive website, where users can report and observe tick sightings on a daily updating interactive map. Punkkilive has been running since April 2021 and has obtained over 220 000 tick observations during this time. While the campaign can be kept running nearly indefinitely due to it requiring little input from researchers, the obvious drawback is that the tick species present at each location cannot be identified.

While crowdsourced data of tick contacts generally likely depict where ticks are present, there are some uncertainties related to this method of acquiring presence data. Firstly, there is the question of whether people participating can correctly identify a tick. Likely due to high media coverage regarding ticks and tick-borne diseases, as well as the (locally) rather unique feeding habits of ticks (being found attached on humans and pets), they are generally well identified in Finland. In the crowdsourcing study in 2015, “almost all” samples were reported to be ticks (Acari: Ixodidae)^20^, whereas for 2021 Punkkilive data, 97.2% of pictures (n = 5573) sent to the website represented ticks, whereas only 1% were of identifiably other animal species. Secondly, since ticks being observed are not often detected in the nature but rather on a host (human, dog, cat)^20^, the precise location from where the tick was acquired is often uncertain. However, this problem can be mitigated by widening the scale at which phenomena are observed, although this procedure sacrifices precision. Finally, another potential issue is that ticks may be transported long distances on e.g. dogs, prior to being detected by humans. Thus, even when the provided coordinate data is precise, the tick locations reported by citizens participating in crowdsourcing campaigns may not in fact reflect the actual areas from where ticks were acquired and/or areas where tick populations are present. In order to mitigate the effects of such imported ticks, assessments need to be made regarding when it is deemed unlikely that all observations could be of such imported ticks.

Despite some drawbacks regarding crowdsourced data, they are nevertheless valuable in depicting in which areas, when and how frequently people and pets are contacting ticks. Likewise, they facilitate the nationwide mapping of tick occurrence, which could not be accomplished with field studies alone. In the current paper, we use three different, nationwide, crowdsourced data sets to assess changes in the geographical distribution of tick contacts in Finland between 1958 and 2021. Likewise, we assess geographical differences in tick contact rates.

## Results

**Figure 1:**
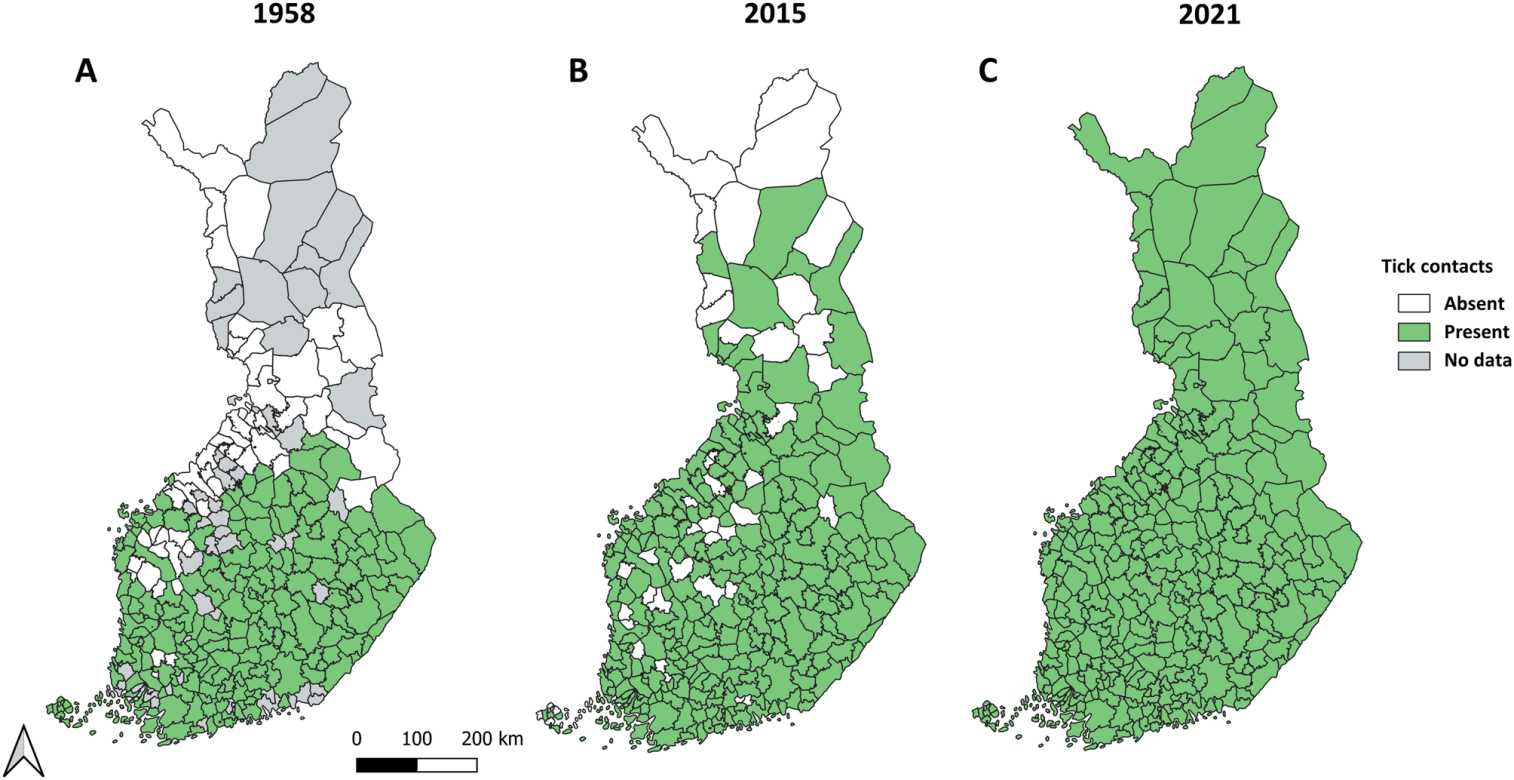
Crowdsourcing-based tick observations in Finnish municipalities (n = 309) in 1958, 2015 and 2021. In 1958, municipalities from which no answers to the sent questionnaires were received got the status “No data”.

**Figure 2:**
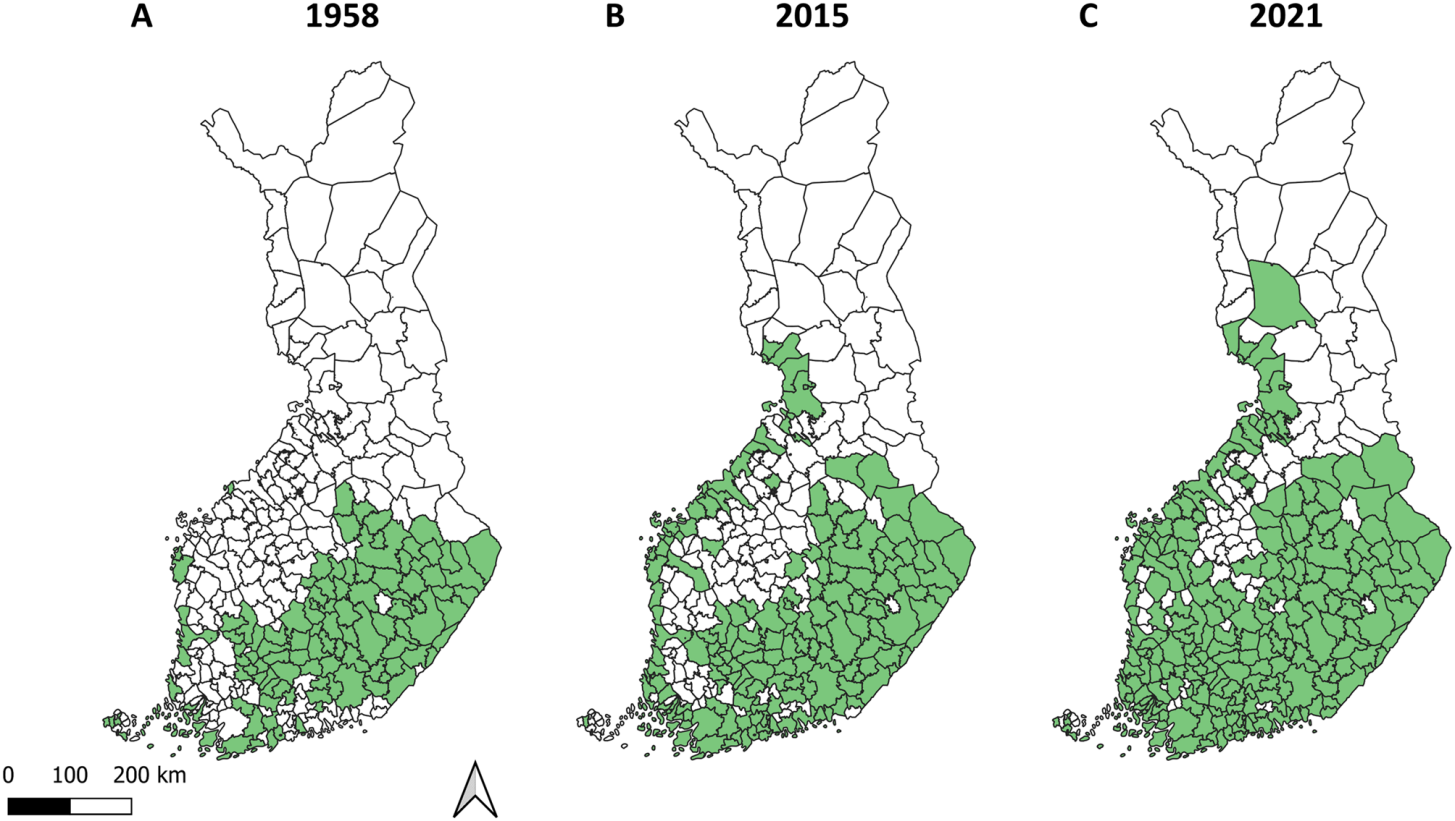
Estimated local tick contact areas (LTCAs) in 1958, 2015 and 2021. Municipalities where at least some of the reported tick contacts were estimated to be locally acquired (forming LTCAs) are colored green.

**Figure 3:**
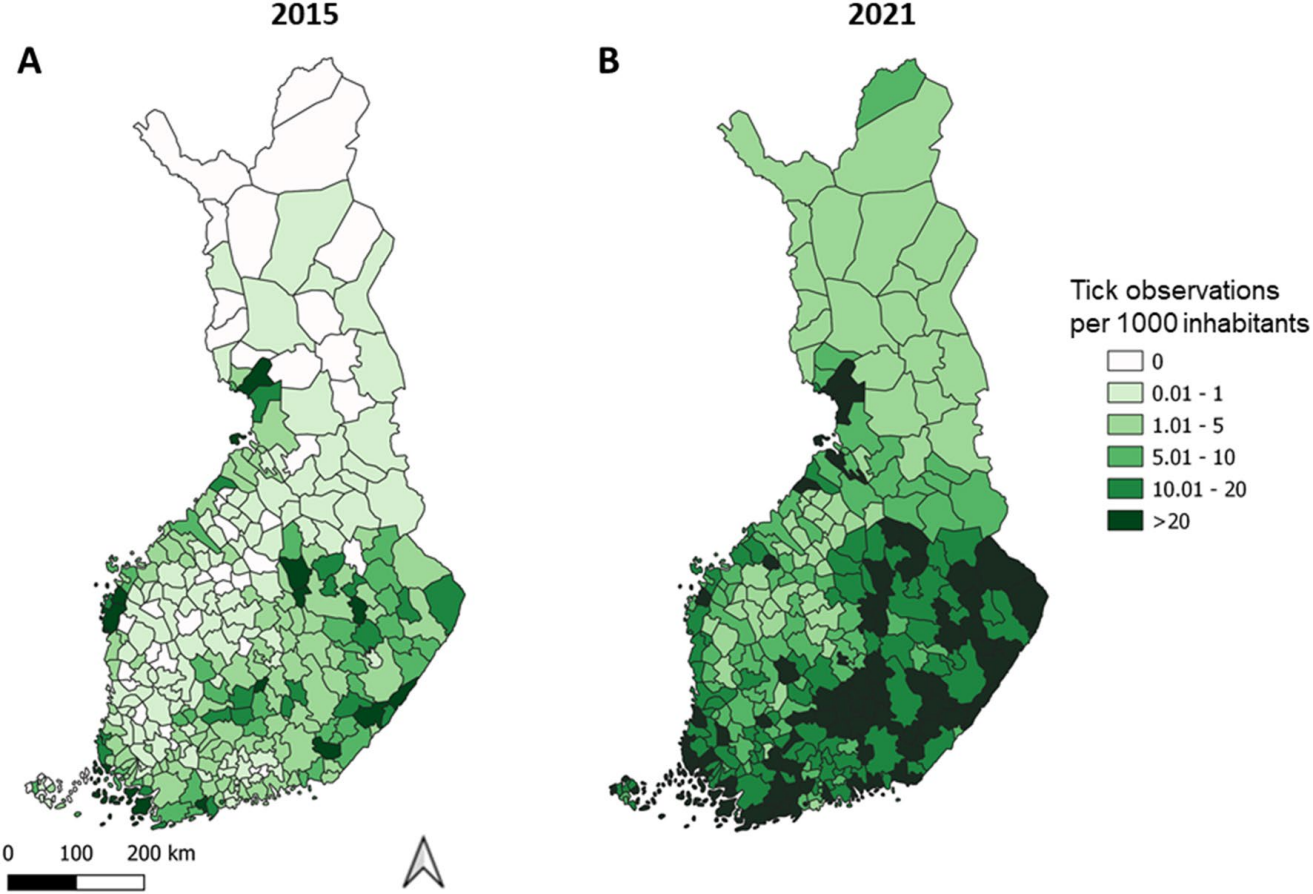
Tick observations per one thousand inhabitants in the crowdsourcing studies in 2015 and 2021. Data presented on the municipality level.

**Figure 4:**
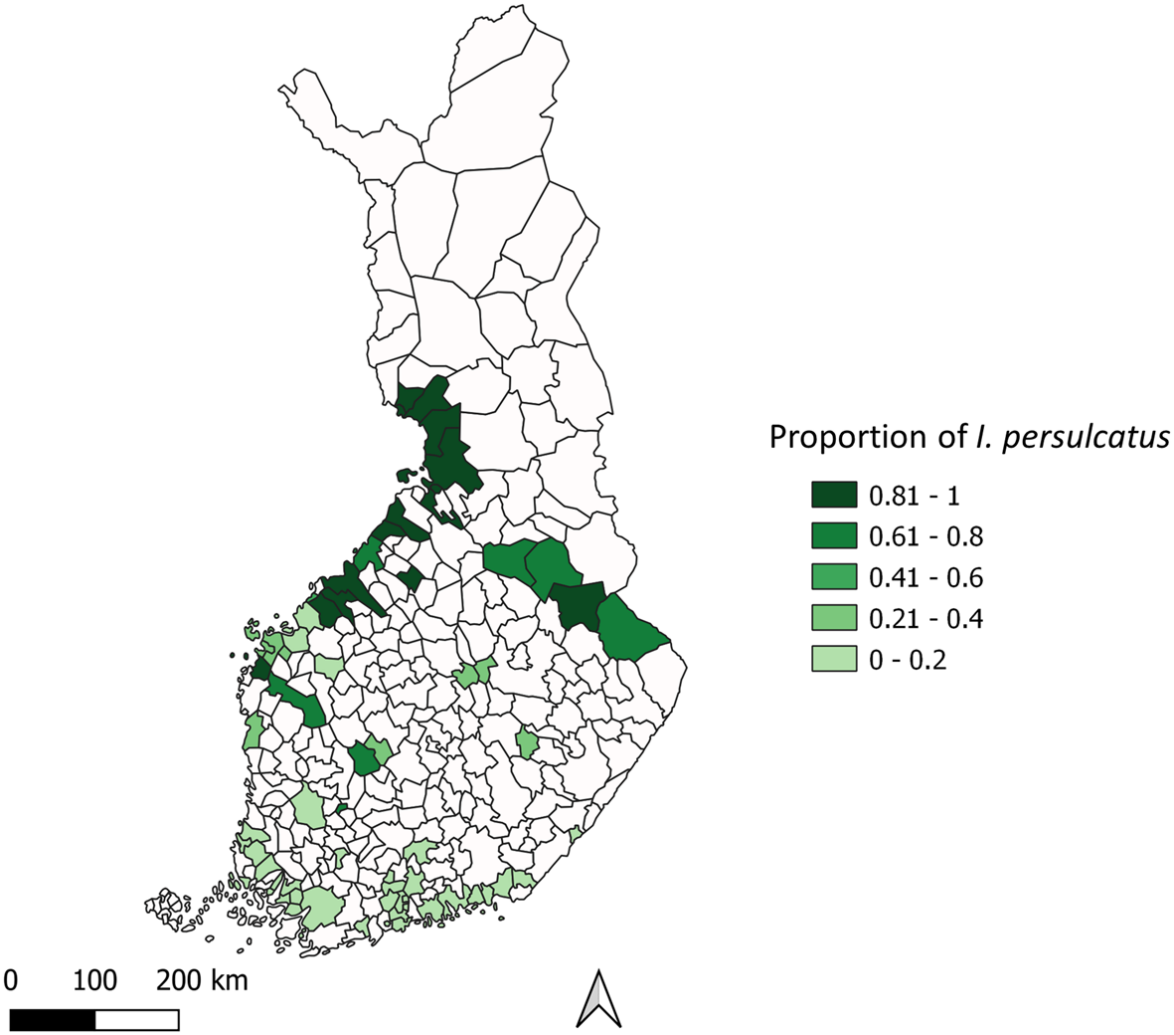
Proportion of *Ixodes persulcatus* in crowdsourced tick samples from 2015 at the municipality level. Included are only municipalities that were classified as local tick contact areas in 2015 but not in 1958.

Tick contacts were reported from 195 out of 309 municipalities in 1958 (63.1% of all; data missing for 60 municipalities), from 261 (84.5%) municipalities in 2015, and from all municipalities in 2021 (Fig. 1). In total, 112 (36.2%) municipalities in 1958 were estimated as areas where at least some ticks were contacted locally (local tick contact areas, LTCAs; see Materials & Methods for details of LTCA classification), 165 (53.4%) municipalities in 2015, and 221 (71.5%) municipalities in 2021 (Fig. 2). The most obvious changes in the spatial distribution of nationwide tick contacts were observed along the Bothnian Bay in western Finland, where several municipalities reporting no tick contacts in 1958 were classified as LTCAs and had high contact rates in both 2015 and 2021 (Figs. 2 and 3). In western Finland, LTCAs extended approximately 400 km further north in 2021 than in 1958. A less pronounced northwards shift could also be observed in eastern Finland, with LTCAs extending approximately 100 km further north in 2021 than in 1958. The northernmost LTCA appears to be Rovaniemi (66° 30′ N, 25° 44′ E) (Fig. 2C). In the southern and central parts of Finland, the numbers of LTCAs increased from 1958 to 2015, as well as from 2015 to 2021 (Fig. 2). In most of the new LTCAs in northern Finland, the proportion of crowdsourced ticks being *I. persulcatus* was between 0.61 and 1, indicating a dominance of this species (Fig. 4). *Ixodes ricinus* mainly contributed towards new LTCAs in southern and central Finland (Fig. 4).

Municipalities from where at least five tick observations per 1000 inhabitants were reported increased from 77 in 2015 to 253 in 2021, reports of at least ten observations per 1000 inhabitants from 40 to 176, and reports of over twenty observations per 1000 inhabitants from 17 to 95. Tick contact rates appeared to be highest along the Baltic Sea coastline (and islands), as well as in proximity to large inland water bodies in southern, central and eastern Finland (Fig. 3).

## Discussion

The nationwide, crowdsourced tick observation data used here suggest a significant expansion in tick contact areas in Finland between 1958 and 2021. In the Punkkilive data from 2021, reports of tick contacts were received from all the Finnish municipalities, whereas in 1958 participants still reported no ticks from many municipalities, most notably on the western coastline approximately north of latitude 63°50′N^17^. The establishment of *I. ricinus* and/or *I. persulcatus* in some municipalities on the western coast is well-known, with some areas from where no ticks were reported in 1958 having been classified as risk areas for TBE during the past few decades^16^. Likewise, field surveys have revealed tick populations in some of these municipalities more recently^21–23^. However, while a general expansion in tick contact areas seems apparent, tick contacts were also reported from the northernmost municipalities in Finland in 2015 and 2021, where the occurrence of established local tick populations is not likely due to short vegetation periods (< 130 days)^5,11,24^ (Fig. S1). Likewise, field surveys over several years have revealed no local ticks in four of these northern municipalities^11^. These reported northernmost tick contacts are most likely to represent ticks that were transported from more southerly areas on humans or dogs. The impact of such imported ticks needs to be taken into account in order to more reliably assess where ticks are contacted locally and, consequently, where local tick populations may be present.

In order to mitigate the effect of imported ticks and achieve a better understanding of nationwide tick occurrence, an attempt was made to determine the areas where at least some observations are likely to be of locally acquired ticks. This was rather straightforward for 1958 data but required the assessment of suitable cutoff numbers of observations for the quantitative data from 2015 to 2021. While cutoff estimation was based on northern municipalities where established tick populations are very unlikely to occur, the chosen values appeared to preserve also other geographical trends in tick occurrence in Finland. Firstly, no LTCAs were estimated in several municipalities situated on the Suomenselkä drainage divide (from SW/SSW to NE/NNE; see figures) in southwestern Finland^20^. Suomenselkä is characterized as a barren area with pine barrens (*Pinus sylvestris*), swamps, and few large water bodies, suboptimal for ticks. Concurrently, tick abundance may be expected to be low and the presence of established populations uncertain. Secondly, the observation of some municipalities in southwestern Finland where reports due to solely imported ticks could not be ruled out in 2015 and 2021 is in line with observations from field studies conducted therein. In field studies in 2014, no ticks were found from five inland study sites in southwestern Finland (transect extending NE from the city of Turku), whereas tick densities were observed to be only 0.13/100 m^2^ at another five inland study sites (no larvae found; ticks found from 2/5 study sites; transect extending N from Turku)^13^. Likewise, in 2021, field surveys in inland study sites proximate to deer feeding stations revealed low tick densities and no larvae, even in the presence of high densities of white-tailed deer (*Odocoileus virginianus*) or roe deer (*Capreolus capreolus*)^25^. Therefore, ticks appear to be uncommon in these areas and the status of established tick populations uncertain. The fact that these observably low tick abundance areas were left without LTCA classification suggests that the classification criteria was not too lenient. Consequently, areas with low observed or expected probability of tick occurrence, such as the northernmost municipalities of Finland in this case, may be suitable for calibrating results from crowdsourcing studies to exclude observations of imported ticks.

The emergence of LTCAs along the length of the western coastline is the most apparent change between 1958 and 2021, with an estimated northwards expansion of approximately 400 km. Some more subtle northward shifts in LTCAs were also observed in eastern Finland. Most of these latitudinal changes occurred between 1958 and 2015. Changes in LTCAs are likely to reflect changes in the abundance and/or occurrence of tick species frequently attaching to humans or pets, in Finland namely *I. ricinus* and *I. persulcatus*^20^. The shifts observed in the geographical distribution of tick contacts are in line with reports of corresponding longitudinal shifts in the geographical distribution of *I. ricinus* in neighboring Sweden and Norway^7,8^. However, unlike in these countries, *I. persulcatus* appears to have also established itself in Finland at some point between 1958 and 2015^17,20–22,26,27^. For this species, there are reports of northwards shifts in geographical distribution from the European parts of Russia^9,10^. In the current study, *I. persulcatus* appeared to be the dominant species in new LTCAs observed in 2015 in the northern parts of Finland, with *I. ricinus* contributing mostly to new LTCAs in southern and central Finland. Consequently, it is possible that that the majority of the new tick contact areas observed in western, eastern, and northern parts of Finland are due to west-northwestwards expansion of *I. persulcatus*, rather than northwards expansion of *I. ricinus*^20^. The expansion of the geographical distribution of *I. persulcatus* appears to still be ongoing, as highlighted by the first reports of the species from the coast of Ostrobothnia in Sweden in 2015, close to the border of Finland^24^. Based on studies regarding habitat suitability^28,29^ and observations of *I. persulcatus* further south in Estonia and Latvia^30,31^, there are likely no abiotic factors limiting their further spread south in Finland or along the Bothnian Bay in Sweden.

The most apparent changes between the years 2015 and 2021 are the increase in LTCAs in southern Finland and the apparently recent establishment of a LTCA in Rovaniemi, consequently likely indicating the northernmost tick population in Finland (Fig. 2C). While only a few individual reports of tick contacts were received in 2015^20^, over 100 tick observations have been reported annually to Punkkilive from the municipality of Rovaniemi in 2021–2023 (unpublished own data). Consequently, it appears probable that ticks are being contacted locally and consistently. The majority species present remains undetermined: in 2015, only a few tick samples were received from the area, and both *I. ricinus* and *I. persulcatus* were recorded^20^. In any case, the spread of either tick species further north seems unlikely at this time due to the shortness of vegetation periods beyond Rovaniemi. However, greater than average warming has been observed in the subarctic in recent decades, so changes enabling further spread may occur sooner than anticipated^32^.

It should be highlighted that different methodologies were used to estimate LTCAs: in 1958, a single report from a person stating ticks to be common in the municipality was sufficient for the status (qualitative data), whereas several such observations would have been required in 2015 and 2021, as assessment was based on observation counts (quantitative data). It is clear that these methods of assessment are not fully comparable, and it is difficult to estimate which might be more reliable. In general, the more observations there are from an area, the more likely it is that at least some ticks were acquired therein. However, there are no universal guidelines to determine cutoff points above which reports from citizens may be expected to represent local tick populations. In this case, assessments of these cutoffs were based on several northern municipalities, where the occurrence of local tick populations is very unlikely. However, it is unclear how well trends relating to observation numbers and observers in the north are reflected in more southern municipalities, with generally higher population density. As for the 1958 data, municipality-level assessments were mostly based on single answers to a questionnaire, so they are highly subjective. A single respondent may have been reporting only personal observations made in nature during one summer or, in case of veterinaries, observations from several animals and over several years, leading to differences in the extent and reliability of assessments. Nevertheless, while keeping in mind these uncertainties regarding the data, it is worth remembering that even a single observation from an environmentally suitable location can indicate a local tick population. As such, the chosen method of assessing LTCAs may be considered rather conservative and more prone to underestimation than overestimation.

Tick contact rates appeared to be highest along the coastline and on the shores of large lakes in southern, central and eastern parts of the country, possibly indicating higher tick abundance therein. Indeed, field studies conducted along the coastline and on islands in southwestern Finland have indicated higher tick abundance compared to e.g. inland areas not proximate to large water bodies^11,13,25,27^. Likewise, similar trends in spatial distribution have been observed regarding the incidence of Lyme borreliosis in Finland^14^. From 2015 to 2021, estimated tick contacts per 1000 inhabitants generally rose, but mainly maintained the overarching structure of lower contact – higher contact areas, wherein higher contact rates were reported along the coastline and the large inland lakes. While longitudinal studies are largely missing from Finland, there has been some indication of increasing tick abundance in the studies conducted^11–13,33^. Also supporting increasing tick abundance across the country, the numbers of diagnosed borreliosis and TBE cases have been increasing during the past few decades^14–16^. Finally, the University of Turku Tick Project (www.puutiaiset.fi) has received many testimonies from citizens claiming new occurrence or higher tick abundance around houses/summer cottages during the past decade (unpublished own data). Consequently, there appears to be an increasing trend in tick occurrence and abundance across the country. However, it is uncertain as to whether this could explain such changes in tick contact rates between 2015 and 2021 – likely, other factors are involved.

Indeed, it is important to highlight that assessing tick abundance based on observations per inhabitants (or crowdsourced data in general) only forms a very tentative proxy, as several factors can influence results apart from tick abundance itself. In the context of the crowdsourcing studies in 2015 and 2021, these factors include higher media coverage and awareness of the study in 2021 (leading to a higher percentage of the population being aware of the study and participating), increased awareness of ticks (leading to more observations), ease of participating (sending ticks via letter in 2015 vs electronic reporting via smart phone in 2021), and changes in outdoor activity of humans (more people doing outdoor activities in 2021 due to COVID-19 restrictions regarding indoor spaces and travel abroad). Another possible source of error are summer visitors to cottages. Several municipalities in Finland have more summer cottages than permanent residences (63 out of 309 municipalities; data from 2020 from Statistics Finland). Summer cottages tend to be located on the shores of the Baltic Sea and lakes, i.e., the areas where most tick observations are made. As summer visitors are not included in national censuses, observations made by them may cause overestimation in relation to number of inhabitants. However, summer visitors commonly contacting ticks also serves as an indication of high local tick abundance, even if the value per inhabitants may be inflated. And again, based on apparently increasing tick numbers and Lyme borreliosis cases in Finland^11–14^ and several reports from citizens of increasing tick densities (unpublished own data), increased contacts due to higher tick abundance and/or new areas of tick occurrence remain plausible explanations. In any case, assessments regarding tick abundance based on crowdsourced data have to be made and interpreted with extreme care.

In conclusion, crowdsourced tick contact data from across 60 years has revealed major increases in tick contact areas in Finland. Both *I. ricinus* and *I. persulcatus* are common in Finland and have been expanding their geographical ranges in the past decades^7–10^. However, the high proportion of crowdsourced ticks from new LTCAs in the northern parts of Finland being *I. persulcatus* suggests that they may be behind most of the increases in tick contact areas in eastern, western and northern Finland^17,20^. Observations of increased tick contact areas are generally in line with local study results regarding tick abundance^11–13^ and tick-borne disease cases^14–16^, which have indicated increasing trends during the past few decades as well. While ticks appear to now be present in most of Finland, there are still areas where tick abundance is low and/or establishment not possible, mainly in northern Finland. Results regarding tick contact rates suggest that contacts are most common in popular summer cottage areas. As a further step in the prevention of tick contacts and infections with tick-borne pathogens, it might be prudent to increase tick awareness in rental companies and property owners of summer cottages, particularly when the cottages are commonly rented out. These actors should highlight the possibility of tick contacts in the vicinity of the rented properties in risk areas, as summer visitors renting these cottages may not realize they could be at risk.

## Materials and methods

### Research data

Three crowdsourced data sets were used to assess the spatial distribution of tick contacts in Finland at different time points: 1958, 2015 and 2021. In the context of this manuscript, all observations from these studies are considered “tick contacts”, i.e. recorded contacts (physical or visual) between ticks and humans, dogs, cats or other domestic animals. No attempts were made to differentiate *I. ricinus* and *I. persulcatus*, the two human-biting, generalist tick species present in Finland, in the context of this study ^20^.

Data containing observations from between 1910 and 1958 was obtained from research published in 1961^17^ (henceforth “1958 data”). In the paper, a questionnaire regarding the occurrence of ticks was sent by letter to approximately 500 people in Finland, mainly veterinaries and subscribers of the biologically oriented scientific magazine “Luonnon Tutkija” (published by Societas Biologica Fennica Vanamo). The author notes receiving “somewhat more than 200 replies”^17^. Tick occurrence was assessed as common, uncommon or lacking, based on the subjective assessments of the participants. The data is presented as a list of Finnish municipalities and icons depicting common, uncommon or lacking status for ticks therein. Furthermore, areas with no responses to the questionnaire are indicated by no icon. Many changes in municipalities have occurred since 1961, with several merges and some splits. The data was reclassified to match current municipalities (from 2021). We gave the highest rating for tick occurrence (order: common, uncommon, lacking) found in the municipalities being merged. For example, if municipalities A, B and C (with common, uncommon and uncommon statuses respectively) merged, the new merged municipality would receive the value “common”. In case a municipality was split into two or more municipalities, all new municipalities received the value of the split municipality.

Data for 2015 was obtained from a crowdsourcing campaign conducted by the University of Turku Tick Project^20^. During the campaign, people were asked to send ticks they found from themselves, pets or domestic animals by letter to the Zoological Museum at the University of Turku. At the start of the campaign, the Tick Project prepared a press release to promote it. Following the press release, information regarding the campaign was spread throughout various national media and the campaign was highly visible throughout the summer. Approximately 7000 letters, containing 20 000 ticks, were received during the campaign. Coordinate data was obtained manually by biologists at the University of Turku, based on information included in the letters^20^. The data has been used in several publications concerning ticks and tick-borne pathogens and diseases^16,19,20,28,29^.

Data for 2021 was obtained during the first year of action of an interactive website for monitoring tick risk areas and activity in Finland, Punkkilive (www.punkkilive.fi/en). On the website (optimized for smart phone use), people can report tick sightings on an interactive map and observe where reports of ticks have been made. The website was launched in April and extensively promoted by press releases and interviews in different media in 2021. In total, approximately 78 000 tick observations were reported to the service during April-December 2021.

### Utilization of data

Tick observation data was used to assess the geographical extent of tick contact areas and variation in tick contact rates. Assessments were made on the municipality level (municipality list from 2021), as 1958 data was only available on that scale.

The extent of tick contacts was assessed by transforming data from each year to simple presence/absence data. In addition, municipalities with missing data (no observers) from 1958 were classed as ‘missing data’. For data from 2015 and 2021, no differentiation between areas with no ticks or no observers was possible (presence only data). As information about the crowdsourcing campaigns was far-spread in both 2015 and 2021, we considered these areas collectively as absence areas.

However, as outlined earlier, crowdsourced tick observations are likely to include not only locally acquired, but also imported ticks. In order to make a more precise assessment of where ticks can be locally acquired due to a local, viable tick population (henceforth known as “local tick contact areas”, LTCAs), we wanted to exclude areas where there is a palpable risk of all observations being of imported ticks. For 1958 data, we used the tick occurrence status “common” to signify LTCAs. For 2015 and 2021 data, we needed to estimate cutoff values for observation numbers, above which the probability of only imported ticks may be expected to be small. As a guideline, we used several municipalities in northern Finland, where the occurrence of established tick populations is very unlikely and observations likely solely of imported ticks, due to:

1. Field surveys, that have over several years revealed no ticks from four such municipalities^11^ (Figure S1).
2. Vegetation periods < 130 days. Established populations of neither *I. ricinus* nor *I. persulcatus* are expected at such areas^5,24,34^ (Figure S1).

Due to uneven numbers of observations (7 000 in 2015 vs. 78 000 in 2021) and different reporting methods (physical letters vs. electronic reporting), estimations were made separately for 2015 and 2021 data. Assessment was done manually, checking maximum values of observations from each municipality belonging to the above categories (n = 13) (Fig. S1). The maximum numbers of observations from these municipalities were 6 in 2015 and 26 in 2021. A total of 140 (standard deviation of observation numbers: 1.9) of all municipalities in 2015 and 78 (standard deviation: 6.8) of all municipalities in 2021 had equal or lower observation numbers than the northern municipalities in their respective years. To add some buffer to the cutoff values, the respective standard deviations were added to the maximum observation numbers, arriving at cutoffs of > 7 for 2015 and > 33 for 2021 (data in integers). Above these cutoffs, at least some observations were estimated to be locally acquired, forming LTCAs.

While there was a possibility to use data on observations per citizen for the above purpose, we refrained from doing so. The aim was to estimate, within the confines of each crowdsourcing study, the maximum number of observations that could conceivably be of only imported ticks (observations from people/companion animals visiting from elsewhere/having visited elsewhere). Since each municipality is unique in regard to how commonly they are visited by outsiders and how often local inhabitants visit other municipalities, the rate between visitors and inhabitants is likely to vary (for example, 63 Finnish municipalities have more summer cottages than permanent residences; 2020 data from Statistics Finland). Furthermore, since particularly the northern municipalities have low population density but are popular areas to visit for many outdoor activities (e.g., hiking and fishing), observations per inhabitant are commonly inflated beyond being useful. This is best demonstrated in the northernmost municipality, Utsjoki, in 2021 (Fig. 3), where only 7 observations were sufficient to raise observations per 1000 inhabitants to nearly 6 (1176 inhabitants). Finally, regarding other possible measures, classification by percentiles and similar measurements impose a restriction on the number of municipalities which can be either LTCAs or not, which is not biologically supported – there is no limit to how many municipalities need to have or not have local tick populations.

In order to assess species-specific contributions of *I. ricinus* and *I. persulcatus* towards new LTCAs, we calculated the proportions of crowdsourced ticks in 2015 being *I. persulcatus* in each of the new LTCA municipalities. Calculations were only made for municipalities newly classified as LTCAs in 2015 (i.e. 1958 vs 2015), as no tick species data are available for 2021.

For assessments of tick contact rates, we calculated the numbers of observations per 1000 inhabitants on the municipality level for the 2015 and 2021 data. No such assessments could be made for 1958 data, and the map of LTCAs was created to match areas where ticks were reported to be common. Population census data on the municipality level from 2015 and 2021 were used for their respective years (data from Statistics Finland). Raw count data of observations on the municipality level is presented in Figure S2.

### Supplementary Information

Supplementary Figure S1.

## Data Availability

The datasets (2015 and 2021) analyzed during the current study are not publicly available due to the raw coordinate data enabling the identification of individual households/properties/etc. from which ticks have been detected (and, correspondingly, the sender). Municipality-level datasets for 2015 and 2021 data analyzed during the current study are available from the corresponding author on reasonable request.
