# Supplementary material for: Crowdsourced tick observation data from across 60 years reveals major increases and northwards shifts in tick contact areas in Finland
Source: Sci Rep. 2023 Dec 2;13:21274. doi: 10.1038/s41598-023-48744-8 (PMC10693632; doi:10.1038/s41598-023-48744-8)
Supplement: Supplementary file 1 — Supplementary Figure S1. [file 41598_2023_48744_MOESM1_ESM.docx]

**Supplementary Data**

Crowdsourced tick observation data from across 60 years reveals major increases and northwards shifts in tick contact areas in Finland

Jani J. Sormunen, Ilari E. Sääksjärvi, Eero J. Vesterinen, Tero Klemola


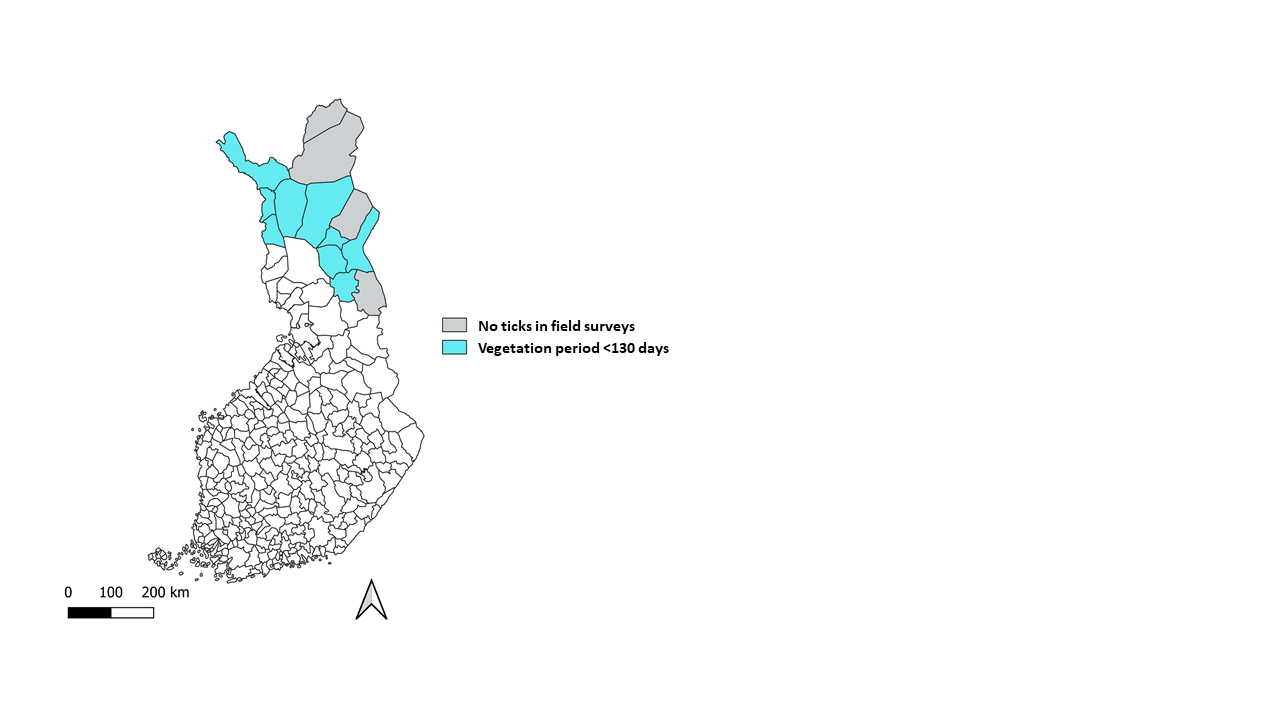
Fig 1S. Finnish municipalities where local tick populations are not expected to occur. All the colored municipalities (n=13) have vegetation periods <130 days. In addition, in four municipalities (grey), no ticks have been found during several years of field surveys [1].


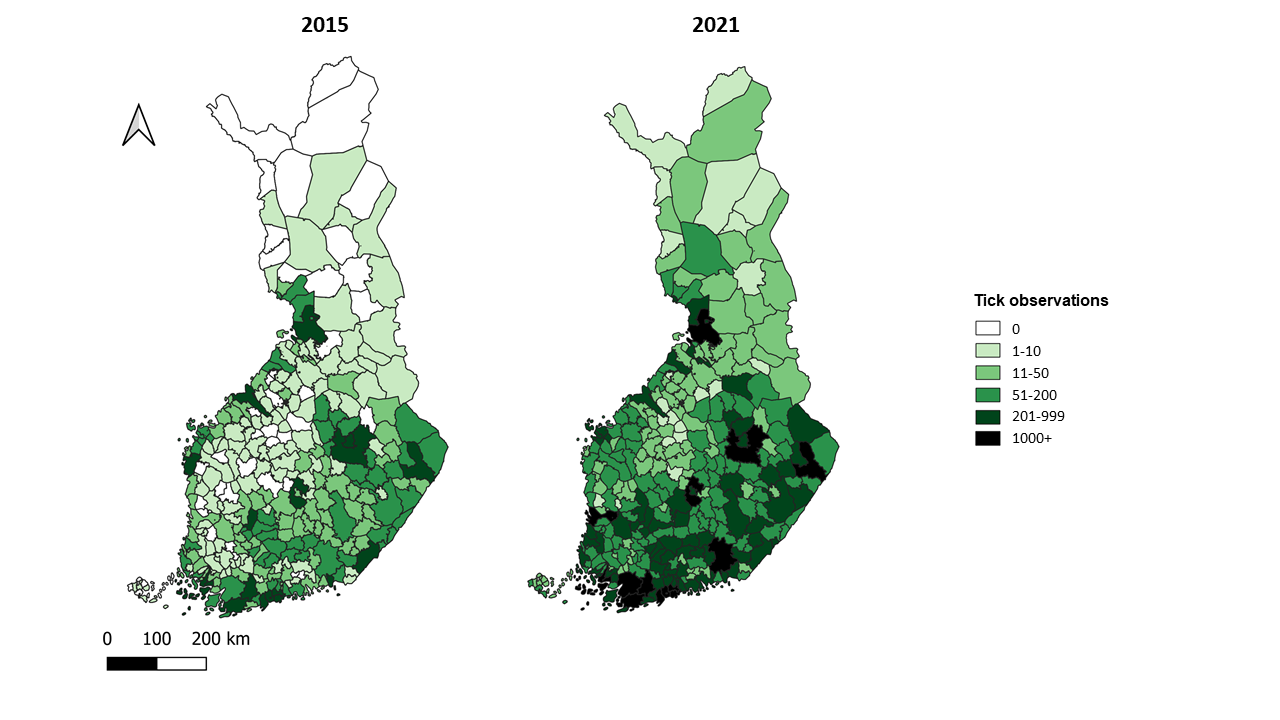


Fig 2S. Absolute numbers of observations received in the crowdsourcing studies in 2015 and 2021, by municipality.

References

[1] J. J. Sormunen *et al.*, "Monitoring of ticks and tick-borne pathogens through a nationwide research station network in Finland," *Ticks and tick-borne diseases,* vol. 11, no. 5, 2020, doi: <https://doi.org/10.1016/j.ttbdis.2020.101449>.
